# Pain in the network of genetic and epigenetic control

**DOI:** 10.1186/1744-8069-10-S1-O9

**Published:** 2014-12-15

**Authors:** Jörn Lötsch, Alfred Ultsch

**Affiliations:** 1 Institute of Clinical Pharmacology, Goethe - University, Theodor-Stern-Kai 7, 60590 Frankfurt am Main, Germany; 2 DataBionics Research Group, University of Marburg, Hans-Meerwein-Straße, 35032 Marburg, Germany

## 

In a significant proportion of patients pain persists despite sophisticated analgesic therapy. In addition, current available treatments provide sufficient pain relief only in a fraction of chronic pain patients. This triggers intensive research and drug development activities. The study of the genetic regulation of pain and its inhibition is thereby considered as a key approach to the development of effective treatments. Inherent parts of this approach are, besides changes in the function of the gene products due to genetic variants, alterations in the expression of pain-relevant genes due to genetic variants at sites relevant for gene transcription, splicing or DNA stability. Moreover, mechanisms of gene expression control, which emerge from several independent lines of contemporary research, point at so far unappreciated complexity of gene expression control exceeding current paradigms. This involves micro RNA control, that according to novel analysis acts on all levels of gene expression including transcriptional fine-tuning, mRNA processing up to translation, which form together with classical epigenetic mechanisms including DNA methylation and histone modifications a regulatory apparatus of pain gene expression. Novel research results clearly indicate that genetics and epigenetics not only affect the pain phenotype and treatment, but the interactions with the genome are bidirectional. Thus, several lines of research evidence point at a high complexity of the genetic control of pain and it is increasingly recognized that this complexity of pain has to be addressed. These developments are accompanied by intensive methodological progresses in modern bioinformatics that increasingly enable the comprehension and utilization of the complexity of the genetic control of pain and its inhibition. Examples of these developments are the exploitations of knowledge base information enabling the identification of relevant biological processes addressed by pain relevant genetic or epigenetic mechanisms. Moreover, analyses of pain phenotypes distributions allow identifying the underlying molecular bases, or may be used to classify pain patients for patterns of symptoms, which facilitates the association of complex pain-relevant genotypes. These developments promise, perhaps for the first time optimistically, a major step toward the understanding of the complexity of pain and the development of effective individualized treatments.

## Disclosures

The authors have declared that no competing interests exist.

